# A Platinized Carbon Fiber Microelectrode-Based Oxidase Biosensor for Amperometric Monitoring of Lactate in Brain Slices

**DOI:** 10.3390/s22187011

**Published:** 2022-09-16

**Authors:** Cândida Dias, Eliana Fernandes, Rui M. Barbosa, Ana Ledo

**Affiliations:** 1 Center for Neuroscience and Cell Biology, University of Coimbra, 3004-504 Coimbra, Portugal; 2 Faculty of Pharmacy, University of Coimbra, 3000-548 Coimbra, Portugal

**Keywords:** lactate, platinized carbon fiber microelectrode, microbiosensor, brain slice, biofouling, potassium-evoked depolarization

## Abstract

Background: Direct and real-time monitoring of lactate in the extracellular space can help elucidate the metabolic and modulatory role of lactate in the brain. Compared to in vivo studies, brain slices allow the investigation of the neural contribution separately from the effects of cerebrovascular response and permit easy control of recording conditions. Methods: We have used a platinized carbon fiber microelectrode platform to design an oxidase-based microbiosensor for monitoring lactate in brain slices with high spatial and temporal resolution operating at 32 °C. Lactate oxidase (*Aerococcus viridans*) was immobilized by crosslinking with glutaraldehyde and a layer of polyurethane was added to extend the linear range. Selectivity was improved by electropolymerization of *m*-phenylenediamine and concurrent use of a null sensor. Results: The lactate microbiosensor exhibited high sensitivity, selectivity, and optimal analytical performance at a pH and temperature compatible with recording in hippocampal slices. Evaluation of operational stability under conditions of repeated use supports the suitability of this design for up to three repeated assays. Conclusions: The microbiosensor displayed good analytical performance to monitor rapid changes in lactate concentration in the hippocampal tissue in response to potassium-evoked depolarization.

## 1. Introduction

L-lactate has received increasing attention in the last years as it has been recognized as a putative energy substrate in brain metabolism while also acting as a signaling molecule [1]. To further clarify the role of lactate in brain metabolism it is essential to develop tools for direct and real-time measurement of lactate concentration dynamics in the brain extracellular space.

In the rodent brain, the extracellular concentration of lactate in vivo is reported to range between 0.8–2.7 mM [2,3,4,5,6]. Lactate concentration dynamics in vivo have been studied in the brain and other tissues as well as cell cultures with several methodologies, including FRET sensors coupled to two-photon microscopy [7], electrochemical measurements [8,9,10,11], mass spectrometry (MS) [12], surface-assisted laser desorption/ionization mass spectrometry (SALDI-MS) [13,14], and liquid chromatography-tandem mass spectrometry (LC-MS/MS) [15]. The use of microelectrodes coupled with fast electrochemical techniques represents an attractive option, as they allow high sensitivity and spatial resolution, with minimal interference and damage to brain tissue [16]. Carbon fiber microelectrodes (CFM) are particularly attractive due to their small size (fibers can go down to 7 µm in diameter), low cost, ease of fabrication, and potential for customization.

The detection of non-electroactive species, such as lactate, can be achieved using microbiosensors based on microelectrodes with an appropriate enzyme immobilized on their active surface. This methodology usually involves the use of oxidase enzymes that produce hydrogen peroxide (H_2_O_2_) as a reporter molecule [17]. Electrochemical detection of H_2_O_2_ is optimal at electrodes comprised of platinum or platinum alloys [18]. Carbon fiber microelectrodes can be used by modifying their surface by deposition of platinum, representing an inexpensive solution. Such microbiosensors provide the opportunity for size reduction compared to conventional platinum wire electrodes, which typically have a diameter of 50–250 µm [19,20].

Rodent brain slices are widely used in neurophysiology studies since they allow control of the extracellular milieu, facilitating variations in medium composition for drug delivery or changing substrate composition, as well as removing limitations imposed by the blood-brain barrier and anesthesia known to impact lactate release in brain tissue [21]. Since several slices can be obtained from the same brain region, multiple conditions may be tested per animal, which allows important refinement to animal experimentation protocols. On the other hand, brain slices retain in vivo cytoarchitecture and intercellular communication and connectivity [22]. As such, brain slices have been widely used in brain metabolism studies [23]. Limitations such as damage to superficial cell layers observed during slice preparation and hypoxia/hypoglycemia at the tissue core due to lack of blood perfusion can be mitigated by fast preparation, use of vibratome instruments, low temperature during preparation, and use of high glucose and oxygen concentrations in the bathing medium used [24,25].

In this work, an electrochemical lactate microbiosensor was designed and optimized for monitoring lactate dynamics in brain slices, at a working temperature of 32 °C, as opposed to 37 °C used for in vivo studies, and using Lactate oxidase (LOx) derived from *Aerococcus viridans*. The analytical performance of the microbiosensors was evaluated in vitro revealing good sensitivity and selectivity, low limit of detection, and operational stability for monitoring changes in lactate concentration in the extracellular space in hippocampal slices. Changes in lactate dynamics were evoked by stimulation with high potassium, revealing a biphasic change in extracellular lactate concentration. Furthermore, we investigated the dependency of the lactate response on glucose supply, supporting the notion that aerobic glycolysis maintains lactate levels in the extracellular space to support energy metabolism under situations of increased neural activity.

## 2. Materials and Methods

### 2.1. Chemicals and Solutions

All chemicals were analytical-grade and purchased from Sigma-Aldrich, Lisbon, Portugal. A carbox gas mixture (O_2_/CO_2_ 95:5) was obtained from Linde, Lisbon, Portugal. All solutions were prepared in bi-deionized MilliQ water with resistivity ≥18 MΩ cm (Millipore Corporation, Burlington, MA, USA). The in vitro evaluation of the microbiosensors was carried out in 0.05 M phosphate buffer saline (PBS) at pH 7.4 with the following composition (in mM): 100 NaCl, 10 NaH_2_PO_4,_ and 40 Na_2_HPO_4_. The 0.4% (*w*/*v*) H_2_PtCl_6_ solution for CFM platinization was prepared in 0.1 M H_2_SO_4_. For evaluation of microbiosensor response, the following stock solutions were used: 1 M lactate prepared in H_2_O, 20 mM ascorbic acid solution in H_2_O prepared freshly on experiment day and a 9.8 mM H_2_O_2_ solution in H_2_O, prepared freshly on the day of the experiment by dilution from a 30% *v*/*v* stock solution.

The medium used for hippocampal slice dissection and recovery period was a modified artificial cerebrospinal fluid (aCSF) with the following composition (in mM): 124 NaCl, 2 KCl, 25 NaHCO_3_, 1.25 NaH_2_PO_4_, 1 MgCl_2_, 1.5 CaCl_2_, 0.2 ascorbate, 1 reduced L-glutathione, and 10 D-glucose. For experiments in hippocampal slice, aCSF with the following composition was used (in mM): 124 NaCl, 2 KCl, 25 NaHCO_3_, 1.25 NaH_2_PO_4_, 1 MgCl_2_, 1.5 CaCl_2,_ and 15 D-glucose. Both were continuously bubbled with carbox for appropriate oxygen supply and to maintain pH 7.4.

### 2.2. Electrochemical Instrumentation

A MultiPalmSens4 (PalmSens BV, Houten, The Netherlands) potentiostat controlled by MultiTrace v. 4.2 software (PalmSens BV, Houten, The Netherlands) was used for fast cyclic voltammetry (FCV) evaluation of CFM general recording properties as well as for electrodeposition of Pt on the surface of CFM. A 3-electrode electrochemical cell was used, comprising the CFM as a working electrode, an Ag/AgCl (3 M NaCl) reference electrode (RE-5B, BASi, West Lafayette, IN, USA), and a Pt wire as an auxiliary electrode. All other amperometric recordings, including in slices, were performed using a 4-channel FAST16mkII potentiostat (Quanteon, Nicholasville, KY, USA) using a 2-electrode electrochemical cell comprising the lactate biosensor/sentinel as working electrodes and either an Ag/AgCl (3M NaCl) (in vitro) or an Ag/AgCl pellet (slice recordings) as a reference electrode.

### 2.3. Lactate Microbiosensors Fabrication

Carbon fiber microelectrodes (CFM) were fabricated as previously described [26]. Briefly, a single carbon fiber (30 μm Textron, Lowell, MA, USA) was inserted into a borosilicate glass capillary (1.16 mm i.d., 2 mm o.d., Harvard Apparatus Ltd., Cambourne, UK) and pulled on a vertical puller (Harvard Apparatus Ltd., Cambourne, UK). The carbon fiber was cut to a tip length of 100–180 µm. Silver paint (RS, Corby, UK) was used to provide the electrical contact between the carbon fiber and a copper wire. The microelectrodes were pretreated and tested for their general recording properties in PBS by FCV at a scan rate of 400 V s^−1^, between −0.4 and +1.6 V vs. Ag/AgCl, for 1 s.

Platinization of the exposed carbon fiber was performed as previously described [20]. Briefly, electrodeposition of Pt nanoparticles was performed using a deoxygenated 0.4% (*w*/*v*) chloroplatinic acid solution in 0.1 M H_2_SO_4_, at a constant holding potential of −0.2 V vs. Ag/AgCl for 10 s.

Lactate oxidase (EC 1.1.3.2, from *Aerococcus viridans* lyophilized powder; LOx) was immobilized at the platinized CFM (CFM/Pt) surface by crosslinking with glutaraldehyde. The CFM/Pt tip was immersed three times in a drop of a mixture of 1 mg mL^−1^ of LOx, 10% (*w*/*v*) bovine serum albumin (BSA), and 0.125% (*v*/*v*) glutaraldehyde in water for 5 min, with 1 min of air drying between dips. The sentinel or null sensors were obtained by following the same procedure, except the enzyme was not added. The sensors were stored in a dry and dark place at room temperature for 72 h for curing. Afterward, a membrane of polyurethane (PU) was added by dipping the sensor tip three times in a solution containing 2% (*w*/*v*) PU in 98% (*v*/*v*) tetrahydrofuran and 2% (*v*/*v*) dimethylformamide for 5 s with 10 min interval (CFM/Pt-LOx-PU). For recording in slices, the sensors were further modified with an exclusion layer of polymerized *m*-phenylenediamine (*m*-PD) on the day of experiments to decrease the interference from oxidation of certain electroactive molecules found in brain tissue (CFM/Pt-LOx-PU-PD). A 10 mM solution of *m*-PD was prepared fresh in N_2_-purged 0.05 M PBS. Electropolymerization was achieved by cyclic voltammetry at a scan rate of 50 mV s^−1^, between +0.25 and + 0.75 V vs. Ag/AgCl for 20 min.

### 2.4. Scanning Electron Microscopy

Microphotographs of the tip of a bare CFM and a CFM/Pt were obtained with high-resolution scanning electron microscopy (SEM) with a field emission scanning electron microscope. The microelectrodes were held on the specimen holder with conductive carbon adhesive tabs. Images were recorded with an acceleration voltage of 25 kV.

### 2.5. In Vitro Evaluation of the Lactate Microbiosensor

Analytical parameters were determined by amperometry at +0.7 V vs. Ag/AgCl in PBS (40 mL) at 32 °C unless otherwise stated.

Enzymatic parameters were determined for the CFM/Pt-LOx-PU microbiosensor by evaluating the response to successive additions of lactate until saturation of response (final concentration range: 0.05–20 mM).

Temperature dependency of the microbiosensor was determined between 30–40 °C by measuring the response to a single addition of lactate (0.5 mM final concentration) at different temperatures. The temperature of the medium was altered by placing the beaker in a circulating water bath and checking the temperature of the media for accuracy. Similarly, pH dependency was determined by measuring the response to a single addition of lactate (0.5 mM final concentration) to PBS at different pH (6.5–8.0).

The sensitivity towards lactate, selectivity against ascorbic acid, and sensitivity towards the reporter molecule H_2_O_2_ were assessed by evaluating the response to successive additions of 0.15 mM of lactate in the presence of 0.1 mM ascorbate followed by a single addition of H_2_O_2_ 0.04 mM. All measurements were performed in the presence of a sentinel sensor.

### 2.6. Preparation of Hippocampal Slices

All animal procedures were performed under the European Union Council Directive for the Care and Use of Laboratory Animals, 2010/63/EU, and were approved by the local ethics committee (ORBEA 146_2016/31102016) and the Portuguese Directorate-General for Food and Veterinary. The animals were purchased from Charles River Laboratory (Barcelona, Spain) and maintained at the local animal facility (Faculty of Medicine, University of Coimbra). Rooms for housing were kept under a controlled environment: a temperature of 22–24 °C, relative humidity of 45–65%, 15 air exchanges per hour, and a 12:12 light/dark cycle. Animals were fed with standard rodent chow and were provided chlorinated water, both ad libitum. Standard corn cob cage bedding was changed three times a week and environmental enrichment was provided with tissue paper and cardboard tubes.

Male Wistar rats (9–12 w.o.) were sacrificed by cervical dislocation under deep anesthesia (isoflurane). After decapitation, the brain was rapidly removed and placed in ice-cold modified aCSF. The hippocampi were dissected and sliced using a vibroslice (World Precision Instruments Ltd., Hitchin, UK) to obtain 400 µm-thick transverse slices. The chamber of the vibroslice was filled with ice-cold modified aCSF continuously bubbled with carbox during slice preparation. Immediately after isolation, the slices were moved to a pre-incubation chamber (BSC-PC, Harvard Apparatus Ltd., Cambourne, UK) containing modified aCSF at room temperature, and continuously bubbled with carbox to maintain an appropriate oxygen supply to the slice core [27]. Slices were allowed to recover for at least 1 h under these conditions.

### 2.7. Experiments in Hippocampal Slices

A single slice was immobilized in an immersion recording chamber (BSC-BU with BSC-ZT top, Harvard Apparatus Ltd., Cambourne, UK), perfused with aCSF continuously bubbled with humidified carbox at 32 °C, at a flow rate of 2 mL min^−1^. All experiments were performed with both the lactate sensor (CFM/Pt-LOx-PU-PD) and a sentinel sensor (CFM/Pt-Null-PU-PD) to ensure that the changes in current were due exclusively to changes in lactate concentration and not due to other electroactive species. The pairing between a lactate biosensor and a null sensor was made based on a similar response to the reporter molecule, H_2_O_2_, and exclusion of the interferent ascorbate. This allowed real-time monitoring of the background and cross-checking of the reliability of the data collected at the lactate biosensor. The lactate microbiosensor was placed in the pyramidal cell layer of the CA1 subregion at a depth of 150–200 µm with the null sensor placed at ca. 100 µm tip-to-tip distance. After stabilization of the background current, the composition of the perfusion medium was changed. The lactate concentration in perfusion was increased by changing the perfusion to aCSF containing successively higher concentrations of lactate (final concentrations in the 0.2–15 mM range). For stimulation, the perfusion was changed to aCSF supplemented with 60 mM KCl for 5 min and then changed back to aCSF.

### 2.8. Evaluation of Lactate Microbiosensor Biofouling upon Successive Slice Recordings

To evaluate putative biofouling of the lactate microbiosensor during recording in slices, each sensor was calibrated as described above (consecutive additions of lactate in the presence of ascorbate, followed by addition of H_2_O_2_) before experiments and following each slice recording. Additionally, we evaluated the effect of submerging the biosensor in brain tissue homogenates on biosensor response to the reporter H_2_O_2_. Homogenates were prepared by homogenizing brain tissue with an ice-cold glass/Teflon potter with 3 strokes. The sensors were placed in homogenates for 2 h at room temperature. The H_2_O_2_ sensitivity was evaluated before and after placing the sensor in the homogenate by determination of the response to a single addition of H_2_O_2_ (final concentration of 0.04 mM) in PBS at 32 °C under gentle stirring. The sensors were then washed in 1% (*v*/*v*) Triton X-100 in PBS under vigorous stirring for 20 min, at room temperature, following which the H_2_O_2_ sensitivity was determined again.

### 2.9. Data Analysis

Data analysis was performed using FAST analysis version 6.0 (Quanteon, Nicholasville, KY, USA), OriginPro 2016 (OriginLab Corporation, Northampton, MA, USA), and GraphPad Prism 5.0 (GraphPad Software, San Diego, CA, USA). For kinetic analysis of the microbiosensors before PD (CFM/Pt-LOx-PU), the data were fitted for each curve to a Michaelis-Menten type equation (GraphPad), allowing the determination of the apparent Michaelis-Menten constant (K_M,app_) for lactate and the maximum steady-state current response (*I*_max_). The sensitivity (slope of the linear regression obtained from the calibration curve, nA mM^−1^) was determined for the linear range up to 0.6 mM. The limit of detection (LOD) was calculated using the following expression LOD = (3 × SD)/m where SD represents the standard deviation of the baseline and m is the slope of the calibration curve.

For the complete microbiosensor used for recordings in slices (CFM/Pt-LOx-PU-PD), lactate sensitivity was determined in the range of up to 0.6 mM. The ascorbate and H_2_O_2_ sensitivities were determined by measuring the current in response to 0.1 and 0.04 mM, respectively. The selectivity against ascorbate was determined as the ratio of sensitivities towards lactate and ascorbate.

### 2.10. Statistical Analysis

Data are presented as mean ± standard error of the mean (SEM). Statistical analysis was performed on GraphPad Prism software. The normality of distribution was evaluated with the Shapiro Wilk test. Statistical significance of differences between two parameters was evaluated by one or two-tailed paired t-test (or Wilcoxon test in the case of a non-normal distribution). Evaluation of comparison between multiple parameters was performed with one-way ANOVA with Holm-Sidak’s multiple comparisons test or Kruskall-Wallis test with Dunn’s multiple comparisons test.

## 3. Results

### 3.1. Analytical Properties of CFM/Pt-Based Lactate Microbiosensors

**Figure 1:**
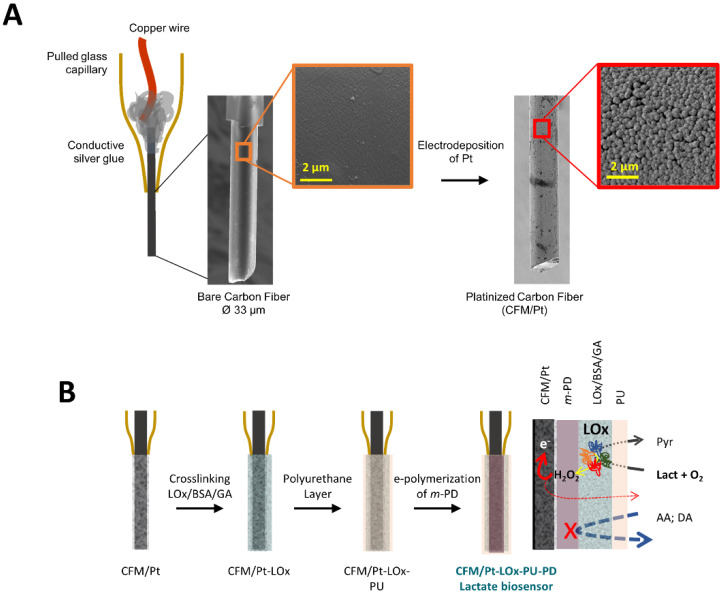
(**A**) Schematic representation of a CFM, showing a scanning electron microscopy (SEM) micrograph of the carbon tip before and after platinization. The blow-up image on the far right shows the surface morphology of the Pt particles on the carbon fiber. (**B**) CFM/Pt-LOx-PU-PD microbiosensor design stages. Starting on the left with a CFM/Pt on which LOx is immobilized by cross-linking with glutaraldehyde in a BSA matrix. The sensor is then coated with a polyurethane layer. In the final step, a permselective membrane of *m*-PD is electropolymerized on the active surface. On the far right is the scheme of microelectrode operation. LOx—lactate oxidase, GA—glutaraldehyde, PU—polyurethane, Lact—lactate, Pyr—pyruvate, AA—ascorbic acid, DA—dopamine.

The active surface of the CFM was first modified by electrodeposition of Pt, resulting in the coverage of the carbon surface with spherical Pt nanoparticles (Figure 1A). As schematized in Figure 1B, LOx was then immobilized on the active surface of the CFM/Pt via cross-linking with glutaraldehyde and BSA to obtain a lactate microbiosensor (CFM/Pt-LOx). A PU membrane was applied over the enzyme layer (CFM/Pt-LOx-PU) to limit lactate diffusion to the enzyme and increase the linear range by increasing the K_M.app_ of the sensor, as previously described [11]. In addition to the LOx microbiosensor, sentinel or null microsensors (CFM/Pt-Null-PU) were built in the same way as the CFM/Pt-LOx-PU, except they lacked LOx. The enzymatic performance was evaluated at this stage, before the addition of the permselective poly-phenylenediamine (PD) layer used to improve the selectivity against ascorbate, as described below. As the PD layer polymerizes at the active surface of the electrode, below the enzyme layer (as schematized in Figure 1B), it has no effect on enzyme kinetics in this configuration.

#### 3.1.1. Kinetic Parameters

**Figure 2:**
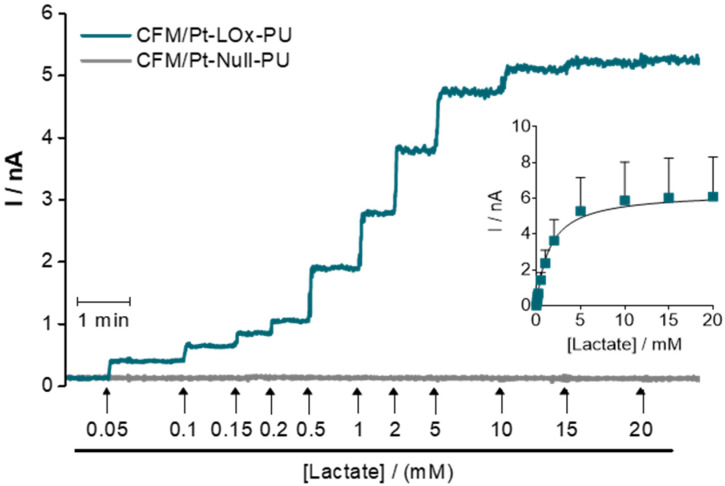
Enzyme kinetics of the CFM/Pt-LOx-PU biosensor. Representative recording showing the response of the CFM/Pt-LOx-PU (blue) and CFM/Pt-Null-PU (grey) sensors to increasing lactate concentrations (up to 20 mM). The addition points and the final concentration of lactate following each addition are indicated by arrows. Inset: Data (current as a function of lactate concentration) was fitted to the Michaelis-Menten equation. Values represent mean ± SEM (*n* = 8).

The analytical properties of CFM/Pt-LOx-PU were tested in PBS pH 7.4, at 32 °C, which is a temperature used for recording in hippocampal slices. It is important to highlight that most other reports in which microbiosensors are designed for in vivo use have performed analytical evaluation at 37 °C. A typical amperometric recording of the response of the sensor and sentinel to successive additions of lactate is presented in Figure 2. The CFM/Pt-LOx-PU displayed a typical Michaelis-Menten behavior with saturation above 15 mM, while de CFM/Pt-Null-PU showed no response to added lactate. The data were fitted to a Michaelis-Menten model, resulting in an average K_M. app_ for lactate of 1.4 ± 0.2 mM and *I*_max_ of 6.8 ± 2.6 nA. From this data, we also determined the mean sensitivity of the CFM/Pt-LOx-PU sensor for the linear range (enzymatic and analytical parameters are presented in Table 1).

#### 3.1.2. Temperature and pH Dependency

**Figure 3:**
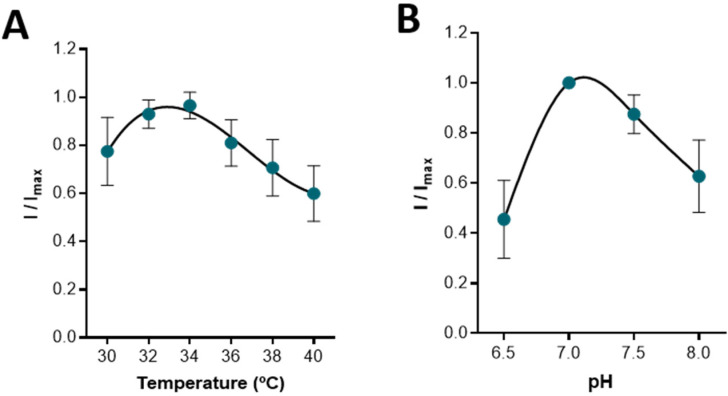
Effect of temperature (**A**) and pH (**B**) on the response of the CFM/Pt-LOx-PU biosensor to 0.5 mM lactate. Data represents mean ± SEM (*n* = 3). For each microbiosensor tested, the current for each temperature or pH value was normalized for the maximal response obtained (*I*/*I*_max_).

Temperature and pH can influence biosensor performance by affecting enzymatic activity, membrane permeability, and oxygen solubility [28]. The response of the CFM/Pt-LOx-PU to a bolus addition of lactate 0.5 mM was measured for different temperatures (30–40 °C) and different buffer pH values (6.5–8.0). As can be observed in Figure 3A, the biosensor exhibited maximal activity between 32–34 °C, which is ideal for recording in hippocampal slices [29,30,31,32]. Regarding pH, the maximal response was observed at pH 7.0, although little change was observed between pH 7.0 and 7.5. Again, this is ideal as the aCSF used in slice experiments is buffered at pH 7.4.

#### 3.1.3. Selectivity

**Figure 4:**
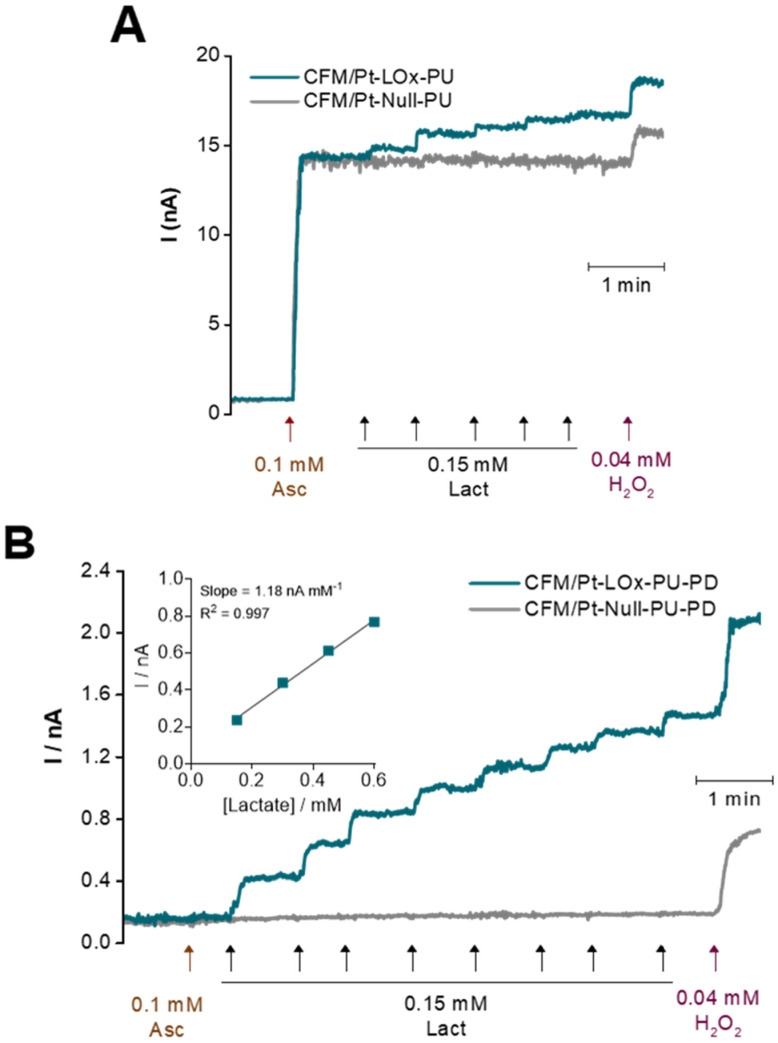
Representative calibration curves of CFM/Pt-based lactate microbiosensors before (**A**) and after (**B**) addition of the PD permselective membrane. Lactate (blue) and null (grey) microbiosensors were calibrated by consecutive additions of lactate in the presence of ascorbate followed by the addition of H_2_O_2_ to evaluate response to the reporter molecule. Additions and their final concentrations are indicated by arrows. Inset in B: Linear regression of the calibration curve up to 0.6 mM.

The major electroactive interferent in the brain extracellular milieu is ascorbate, which is oxidized at a lower potential than H_2_O_2_ and is present at a high basal concentration (0.25–0.5 mM) that can vary in response to glutamatergic neurotransmission [33,34,35]. To mimic conditions in brain slices, the CFM/Pt-LOx-PU microbiosensors were calibrated in the presence of ascorbate. As can be observed in Figure 4A, there was considerable response to 0.1 mM ascorbate, with the mean selectivity ratio (lactate/ascorbate sensitivities) of 0.15 ± 0.06 (*n* = 8). To improve the selectivity of the CFM/Pt-LOx-PU microbiosensor, a permselective membrane of *m*-phenylenediamine (*m*-PD) was added by electropolymerization (CFM/Pt-LOx-PU-PD). The *m*-PD monomers cross the PU and LOx/BSA layers and electropolymerization occurs on the CFM/Pt surface, forming a size exclusion layer that prevents larger molecules such as ascorbate and dopamine from reaching the electrode surface while allowing smaller molecules such as H_2_O_2_ to reach the electroactive surface of the sensor, as depicted in Figure 1B on the right [36,37]. As seen in Figure 4B, this reduced the microbiosensor response to ascorbate, and the selectivity was significantly increased to 5.31 ± 1.37 (*p* = 0.0009, *n* = 6, with two additional sensors presenting no measurable response). Addition of the PD film decreased the sensor response both to lactate (from 2.2 ± 0.2 to 0.6 ± 0.2 nA mM^−1^; *n* = 8) and H_2_O_2_ (from 109 ± 22 to 33 ± 11 nA mM^−1^; *n* = 8). Nonetheless, the lactate sensitivity is still within the desired values for this application. Analytical parameters for the CFM/Pt-LOx-PU-PD microbiosensor are summarized in Table 1.

### 3.2. Evaluation of the Operational Stability of the Lactate Microbiosensor for Multiple Recordings

Others have reported decay of the analytical performance of sensors and biosensors following recording in brain tissue as a result of biofouling and/or damage to the coatings [38,39]. To assess the operational stability of the CFM/Pt-LOx-PU-PD microbiosensors upon repeated use in slices, we evaluated three parameters: sensitivity towards lactate (integrity and functionality of the enzyme layer), sensitivity towards H_2_O_2_ (the integrity of the Pt layer), and selectivity against ascorbate (to assess the permselective PD layer). Microbiosensors were calibrated before use in slices (pre-calibration) and following each recording session in an individual slice. As can be observed in Table 2, a non-significant tendency for a decrease in microbiosensor sensitivity towards lactate was observed up to the fourth slice. However, H_2_O_2_ sensitivity decreased significantly after the second, third, and fourth slices. As for ascorbate sensitivity, it increased only after the third slice, indicating that the PD exclusion layer also remained stable up till then.

The decrease in H_2_O_2_ sensitivity but not lactate was confounding, as H_2_O_2_ is the electroactive reporter produced by LOx. However, while LOx-derived H_2_O_2_ is produced in the enzyme layer immobilized at the CFM/Pt surface, H_2_O_2_ for microbiosensor calibration is added to the bulk solution and must diffuse to the CFM/Pt surface. We hypothesized that the decreased sensitivity to bulk H_2_O_2_ results from the reaction with biomolecules such as proteins and lipids in cellular debris attached to the microbiosensor tip following removal from tissue. To test this hypothesis, sensitivity was evaluated before and after immersion of sensors in brain homogenate for 2 h [38]. The H_2_O_2_ sensitivity was significantly decreased to 63.4 ± 2.8% of pre-calibration values (*n* = 6; *p* < 0.05). Washing the microbiosensor in mild detergent to remove attached debris resulted in a partial recovery of the H_2_O_2_ sensitivity to 75.2 ± 3.3% of pre-calibration values (*p* < 0.01). Thus, we can assume that the decrease in sensitivity towards bulk H_2_O_2_ results from it being consumed in reactions with biomolecules present in the debris which becomes attached to the microbiosensor tip when placed/removed from the tissue.

These results support the use of a single CFM/Pt-LOx-PU-PD microbiosensor in up to three successive slices with no significant loss in analytical performance. As such, the recordings in hippocampal slices presented below were performed using microbiosensors up to three consecutive times, always taking care to calibrate each one before use in each slice to ensure a lactate sensitivity of at least 90% of the pre-calibration value and no significant change in ascorbate selectivity.

### 3.3. Measurement of Extracellular Lactate in Hippocampal Slices

**Figure 5:**
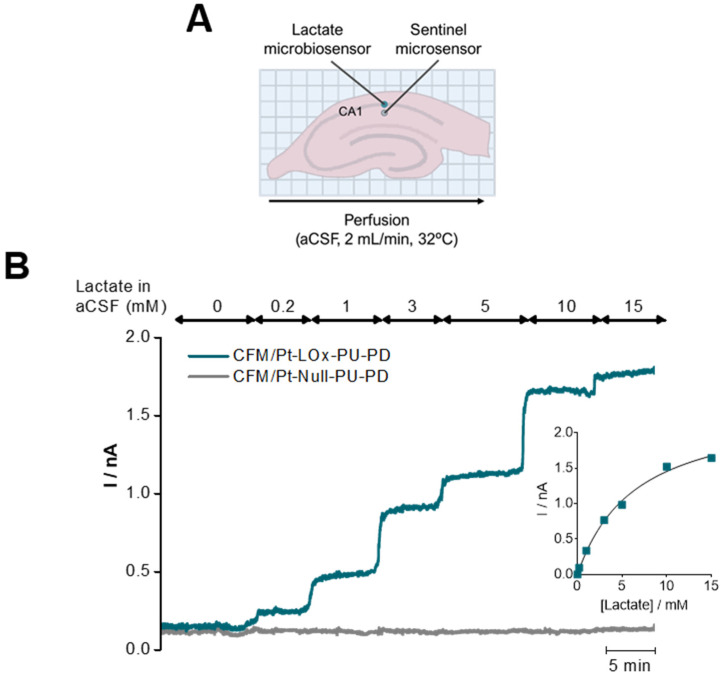
Validation of the suitability of the CFM/Pt-LOx-PU-PD for monitoring lactate in hippocampal slices. (**A**) Schematic representation of insertion sites for both the lactate (blue) and null microsensors (grey) in the hippocampal slice, CA1 subregion. (**B**) Recording of lactate in the extracellular space of hippocampal slices upon incremental increases of lactate in the perfusion media.

To validate the suitability of the lactate microbiosensor to measure changes in extracellular lactate in hippocampal slices, a CFM/Pt-LOx-PU-PD was inserted in the CA1 subregion along with a sentinel sensor, as represented in the scheme of Figure 5A. The concentration of lactate in the perfusion medium (aCSF) was increased in a step-wise fashion from 0 to 15 mM. As can be observed in Figure 5B, incremental changes in lactate increased the current measured by the lactate sensor, while the current from the sentinel sensor remained unchanged. This control experiment was performed in the absence of glucose to discard the contribution of endogenous lactate [40,41,42].

### 3.4. High Potassium-Evoked Changes in Extracellular Lactate in the CA1 Subregion of Hippocampal Slices

**Figure 6:**
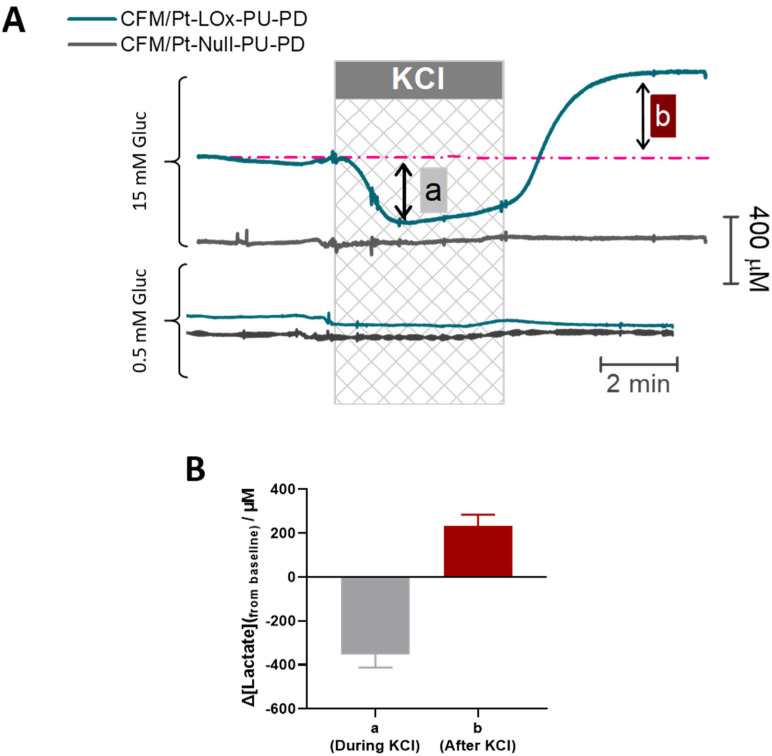
(**A**) Representative recording of extracellular lactate in the CA1 subregion of hippocampal slices upon stimulation with KCl (checkered box, 60 mM, 5 min). The top panel shows a response observed when high glucose (15 mM) is present in the perfusion media while the bottom panel shows a response observed when glucose concentration in perfusion media is low (0.5 mM). (**B**) The plot of the average change in lactate concentration measured in phases a (during KCl stimulation) and b (after KCl stimulation) of each recording.

To validate the suitability of the CFM/Pt-LOx-PU-PD biosensor for monitoring activity-induced changes in extracellular lactate concentration, we used a model of high K^+^-evoked depolarization. This depolarization leads to an increase in energy metabolism to support the activity of pumps and channels that re-establish the ionic gradient [43,44]. As shown in Figure 6A, upon transient stimulation with KCl (60 mM, 5 min), a biphasic change in extracellular lactate concentration was observed. During KCl perfusion (phase a), the lactate concentration decreased, reflecting a net consumption of extracellular lactate by cellular elements. Following KCl removal (phase b), lactate concentration in the extracellular space increased above baseline. After reaching a peak, the lactate tended to slowly recover baseline values. Removal of glucose resulted in a significant decline in measured lactate concentration to a value below the minimum observed in phase a (Figure S1). As shown in Figure 6B, the mean change in lactate observed in phase a was Δ_Lactate_ = −0.36 ± 0.06 mM (*n* = 8) while the mean increase from baseline in phase b was Δ_Lactate_ = 0.23 ± 0.05 mM (*n* = 8).

Considering that in brain slices the oxygen level in the tissue is within physiological levels [27], the main source of extracellular lactate is likely aerobic glycolysis. As such, we asked whether the response to KCl was dependent on glucose supply. When slices were stimulated with KCl in the presence of low glucose (0.5 mM) we observed no measurable change in extracellular lactate (bottom panel in Figure 6A). Production, release, and uptake of lactate by neural cells are fine-tuned by several parameters, but this result highlights the requirement for adequate glucose supply for an activity-dependent flux of lactate in the brain’s extracellular space [42].

Also, worth mentioning is the fact that in both cases, the current recorded at the null sensor did not change, confirming that the signal measured at the CFM/Pt-LOx-PU-PD microbiosensor resulted from lactate.

## 4. Discussion

We have designed and evaluated a microbiosensor based on LOx immobilized on the surface of platinized CFM for monitoring rapid changes in extracellular lactate in hippocampal slices. Although in vivo studies are attractive, the presence of functional cerebral blood flow can hamper the study of the effects of neural response, particularly due to lactate flux. While lactate is found in circulation and can enter brain parenchyma [45], several lines of evidence have shown that this metabolic intermediate is produced by neural cells, most likely astrocytes, and is released into the extracellular space [42]. Brain slices are an attractive ex vivo model for metabolic studies which preserves cytoarchitectural integrity and cell intercommunication while removing confounding effects resulting from blood flow and anesthesia.

The CFM platform is attractive due to its low cost and reduced size, which minimize tissue damage upon insertion into the brain tissue. However, carbon displays poor analytical performance towards the oxidation of H_2_O_2_, the electroactive reporter molecule detected when using oxidase-based biosensors. To circumvent this limitation, we modified the carbon surface by electrodeposition of Pt nanoparticles, as Pt displays excellent properties towards H_2_O_2_ oxidation [18].

Most lactate microbiosensors comparable to that characterized here have been designed for in vivo applications, using a temperature of 37 °C [46,47,48,49], while recording in slice preparations is usually performed at lower temperatures, and in our case at 32 °C [20,27,30,31,50,51]. Additionally, most have used a LOx enzyme obtained from *Pediococcus sp.* while in the present study we have used the currently commercially available LOx obtained from *Aerococcus viridans*. We found that the CFM/Pt-LOx-PU microbiosensor displayed optimal sensitivity between 32–34 °C and for the pH used for slice recordings. One important parameter to consider in the design of biosensors is the K_M.app_ of the immobilized enzyme, considering the expected analyte concentration in the media. The K_M_ of LOx from *Aerococcus viridans* has been estimated to be in the range of 0.12 to 0.94 mM [52,53], which is below the expected basal concentration of lactate in brain tissue. The in vivo concentration of lactate in the rodent brain extracellular space is reported to range between 0.8–2.7 mM [2,3,4,5,6], although in slices it is expected to be lower due to lack of blood flow (we have found the average basal lactate concentration in hippocampal slices to range between 0.3–0.4 mM, data not shown). The CFM/Pt-LOx-PU microbiosensor displayed a K_M.app_ for lactate of 1.4 ± 0.2 mM at 32 °C, above the reported value. This increase is the result of the PU layer, which we and others have shown to impose a diffusional limitation, increasing the biosensor K_M.app_ and linear range to values suitable for measuring lactate in hippocampal slices [11,54,55,56]. Interestingly, using LOx from *Pediocuccus* sp. we were able to increase the K_M.app_ up to 22 mM [56], highlighting different sensitivities of the enzyme from different sources.

Using LOx from *Aerococcus viridans*, we have recently characterized a similar lactate microbiosensor for in vivo applications at 37 °C [11]. For the same design (CFM/Pt-LOx-PU) and using the same enzyme load (0.1%), we found that the decrease in temperature from 37 to 32 °C only significantly decreased the K_M.app_ for lactate (2.5 ± 0.3 mM at 37 °C to 1.4 ± 0.2 mM at 32 °C, *p* < 0.01), while *I*_max_, sensitivity, and LOD remained unchanged and suitable for monitoring lactate in hippocampal slices. While several studies have investigated the effect of temperature on V_max_ (which in the case of an electrochemical biosensor is translated as *I*_max_), fewer have investigated the temperature effect on K_M_, showing that the correlation may be positive, negative, or null, depending on the specific enzyme. Indeed, K_M_ is a measure of the enzyme affinity to the substrate, and variation with temperature is mainly determined by the value of the entropy of dissociation of the substrate [57].

One critical issue in designing biosensors for monitoring chemicals in brain tissue is selectivity against expected interferents, namely ascorbate, which not only can be found in high concentrations in the extracellular space but can also change significantly in concentration upon activation of glutamate neurotransmission [34,35,58,59]. To address this issue, two strategies were used: the application of a permselective membrane composed of electropolymerized *m*-PD and the use of a null or sentinel microbiosensor. We found that *m*-PD effectively increased the selectivity ratio against ascorbate from 0.15 to 5.31, despite the negative impact on lactate and H_2_O_2_ sensitivity. This has been described for similar glucose microbiosensors [20] and it may be due to surface properties modifications [18].

Selectivity criteria are less stringent when working with hippocampal slices as compared to in vivo. The neurons within the hippocampus are mainly glutamatergic and GABAergic, while noradrenergic, serotonergic, and cholinergic terminals derive from afferent pathways primarily originating from outside the hippocampus (the locus coeruleus, raphe nuclei, and septal nuclei, respectively) [60]. As such, while dopamine and serotonin are major concerns for in vivo recordings, in slices, ascorbate is the most relevant interferent [34]. As such, for in vivo recordings shown in [11], the microbiosensor had to be coated with an additional layer of Nafion^®^ for improved selectivity. The decrease in sensitivity resulted in having to increase the enzyme load to 0.5%. Thus, for slice recordings, the design presented here is less laborious in construction and has a lower cost in enzyme. The use of a null or sentinel microbiosensor allows continuous monitoring of the reliability of the signal measured at the lactate sensor. The lactate and null sensors are paired in terms of their sensitivity for the reporter molecule H_2_O_2_ and exclusion of the interferent, ascorbate. As such, any similar changes in the current at both sensors would signal changes not derived from lactate [36,55,61].

Other strategies which have been employed to achieve selectivity include using differential normal pulse voltammetry to monitor lactate in vivo. Even though this differential voltammetric technique allows for good chemical identification of the measured analyte, temporal resolution is significantly compromised (less than 1 point per minute) [62]. The use of Prussian blue (PB) modified CFM as a platform for lactate oxidase-based microbiosensors has also been successful [63,64]. This ferric hexacyanoferrate coordination compound acts as an “artificial peroxidase”, allowing the electroreduction of H_2_O_2_ at a potential close to 0 V vs. SCE, minimizing interference by electroactive species such as ascorbate and dopamine [65,66]. However, PB is notoriously unstable at physiological pH and in the presence of high Na^+^, two conditions observed in the brain extracellular space [67,68]. Replacement of PB with its more stable analog ruthenium purple may be an option worth exploring in the future [69].

Decay of the analytical performance of microbiosensor can result from biofouling and/or damage to the coatings [38,39], hindering multiple uses. We evaluated the operational stability for repeated use at three levels: response to H_2_O_2_ (to evaluate integrity the Pt layer integrity), response to lactate (to determine the functionality of the LOx layer), and also the response to ascorbate (as a measure of PD layer integrity). We found that the response of the microbiosensor remained stable towards lactate for up to three repeated uses in slices, as did the selectivity against ascorbate. However, the sensitivity to H_2_O_2_ decreased. Further assessment revealed that cellular debris attached to the microbiosensor compromised the response to exogenously applied H_2_O_2_, as washing with mild detergent partially recovered the response. The decrease in sensitivity towards exogenously applied H_2_O_2_ is most likely the result of the activity of enzymes such as catalase, which rapidly decompose the biological oxidant to H_2_O [70]. Hydrogen peroxide resulting from LOx activity is produced in the enzyme matrix, near the Pt active surface, where it is readily oxidized, therefore not affected by the cellular debris. The maintenance of analytical parameters, most importantly lactate sensitivity and selectivity against ascorbate after the sensors were used in three slices, is likely due to both the PD membrane and the PU coating, as both have been shown to reduce biofouling [36,71,72].

The lactate microbiosensors were shown to be suitable to study lactate dynamics in brain slices, reporting changes in extracellular lactate concentration in hippocampal slices, both upon exogenous and endogenous modulation. We found that bath stimulation with high K^+^ resulted in a biphasic profile, in accordance with what was observed previously in vivo with synaptic potentiation [9]. The complex change in net lactate concentration in the hippocampus observed upon KCl stimulation was also shown to depend on glucose supply, linking glucose metabolism to extracellular lactate flow. The surge in extracellular lactate after stimulation has been attributed to a release from astrocytes [73]. This appears to occur through a chloride channel in an MCT-independent manner. It has been proposed that this channel allows for the release of lactate despite the alkalinization produced by K^+^ that hinders the H^+^-dependent MCT activity [74].

## 5. Conclusions

We have designed a microbiosensor based on lactate oxidase immobilized on the surface of platinized carbon fiber microelectrodes for monitoring rapid changes in extracellular lactate linked to K^+^-evoked depolarization in hippocampal slices. The in vitro characterization revealed a suitable analytical performance, namely high sensitivity and selectivity, low LOD, and also extended operational stability. This novel lactate microbiosensor was shown to be able to report changes in extracellular lactate concentration in hippocampal slices, both upon exogenous and endogenous modulation. The complex change in net lactate concentration in the hippocampus observed upon KCl stimulation was also shown to depend on glucose supply, linking glucose metabolism to extracellular lactate flow. The lactate microbiosensors here described can be used as a tool to study lactate dynamics in brain slices, an attractive ex vivo model for metabolic studies which preserves cytoarchitectural integrity and cell intercommunication while removing confounding effects resulting from blood flow and anesthesia.

## Figures and Tables

**Table 1:** Kinetic and analytical parameters of CFM/Pt-LOx-PU and CFM/Pt-LOx-PU-PD microbiosensors.

Microbiosensor (*n* = 8)	K_M,app_ (mM)	*I*_max_ (nA)	Lactate			H_2_O_2_ Sensitivity (nA mM^−1^)	Selectivity (Lactate/AA)
			Sensitivity (nA mM^−1^)	Linearity (R^2^)	LOD (mM)		
CFM/Pt-LOx-PU	1.4 ± 0.2	6.8 ± 2.6	2.2 ± 0.7	0.994 ± 0.002	0.04 ± 0.01	109 ± 22	0.15 ± 0.06
CFM/Pt-LOx-PU-PD	-	-	0.6 ± 0.2	0.993 ± 0.002	0.07 ± 0.01	33 ± 11	5.31 ± 1.37 *

* *n* = 6.

**Table 2:** Analytical parameters of CFM/Pt-LOx-PU-PD before (pre-calibration) and after each successive recording session in slices are shown as a percentage of the pre-calibration values or in nA mM^−1^ (ascorbate sensitivity). Values represent mean ± SEM. * *p* < 0.05, for comparison to pre-calibration value.

Condition (n)	Sensitivity		
	Lactate (% of Pre-Cal)	H_2_O_2_ (% of Pre-Cal)	Ascorbate (nA mM^−1^)
Pre-calibration (14)	100	100	0.20 ± 0.07
After 1st recording (6)	90.0 ± 9.4	92.8 ± 4.0	0.08 ± 0.08
After 2nd recording (4)	91.0 ± 16.1	74.8 ± 7.4 *	0.12 ± 0.12
After 3rd recording (8)	90.8 ± 5.6	71.4 ± 10.8 *	0.58 ± 0.15 *
After 4th recording (4)	74.0 ± 17.2	61.8 ± 7.5 *	0.69 ± 0.49

## Data Availability

The data presented in this study are available on request from the corresponding author.

## Supplementary Materials

The following supporting information can be downloaded at: https://www.mdpi.com/article/10.3390/s22187011/s1, Figure S1: Lactate measurements in the CA1 subregion of hippocampal slices with bath KCl stimulation (60 mM; 5 min).
