# Supplementary material for: A Platinized Carbon Fiber Microelectrode-Based Oxidase Biosensor for Amperometric Monitoring of Lactate in Brain Slices
Source: Sensors (Basel). 2022 Sep 16;22(18):7011. doi: 10.3390/s22187011 (PMC9501957; doi:10.3390/s22187011)
Supplement: Supplementary file 1 [file sensors-22-07011-s001.zip › sensors-1878473-supplementary.pdf]

Supplementary Materials

A Platinized Carbon Fiber Microelectrode-Based Oxidase Bio-sensor for Amperometric Monitoring of Lactate in Brain Slices.

Cândida Dias[1,2], Eliana Fernandes[1], Rui M. Barbosa[1,2], Ana Ledo[1,2,*]

[1]Center for Neuroscience and Cell Biology, University of Coimbra, Portugal
[2]University of Coimbra, Faculty of Pharmacy, Portugal

*Corresponding author
Ana Ledo
Faculty of Pharmacy and Center for Neuroscience and Cell Biology
University of Coimbra
Health Science Campus
Azinhaga de Santa Comba
3000-548 Coimbra
Portugal
analedo@ff.uc.pt

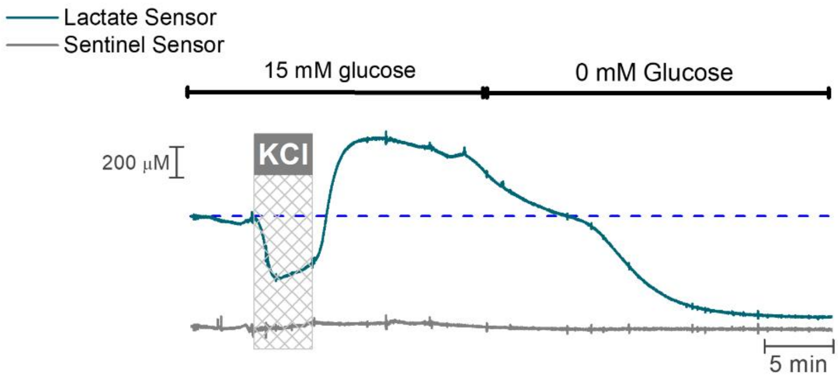

Figure S1 - Lactate measurements with CFM/Pt-based lactate microbiosensors (CFM/Pt-LOx-PU-PD; blue) in the CA1 subregion of hippocampal slices in the presence of a sentinel microsensor (CFM/Pt-Null-PU-PD; grey) with bath KCl stimulation (60 mM; 5 min) in the presence of 15 mM glucose and then with removal of glucose from the perfusion media.
